# Poor glycemic control and associated factors among pediatric diabetes mellitus patients in northwest Ethiopia, 2020: facility-based cross sectional retrospective study design

**DOI:** 10.1038/s41598-022-19909-8

**Published:** 2022-09-19

**Authors:** Atitegeb Abera Kidie, Birtukan Gizachew Ayal, Tiruneh Ayele, Elsa Awoke Fentie, Ayenew Molla Lakew

**Affiliations:** 1 School of Public Health, College of Health Science, Woldia University, Woldia, Ethiopia; 2 Department of Epidemiology and Biostatistics, Institute of Public Health, College of Medicine and Health Science, University of Gondar, Gondar, Ethiopia; 3 Department of Public Health, College of Medicine and Health Science, Wollo University, Dessie, Ethiopia; 4 Department of Reproductive Health, Institute of Public Health, College of Medicine and Health Science, University of Gondar, Gondar, Ethiopia

**Keywords:** Endocrinology, Endocrine system and metabolic diseases

## Abstract

Diabetes mellitus is a global public health problem. Glycemic control is a major public health problem. Diabetes results from elevated levels of glycaemia such as increased glucose and glycated hemoglobin, and controlling glycaemia is an integral component of the management of diabetes. Glycemic control in children is particularly difficult to achieve. Identifying determinants of poor glycemic control is important for early modification of diabetic related end organ damages. This study was aimed to assess the status of glycemic control and associated factors among pediatric diabetes mellitus patients in northwest Ethiopia. Facility-based cross sectional retrospective cohort study design was used and this study was conducted from September, 2015 to February, 2018. Simple random sampling was used to select 389 samples. Data were collected using an extraction checklist. Data were entered into Epi-data − 4.6, and analyzed using Stata-16. Finally, multivariable binary logistic regression was done. Poor glycemic control was more common among pediatric patients 39.3% (95% CI 34.6, 44.3). Treatment discontinuation (AOR 2.42, 95% CI 1.25, 4.69), age (AOR 1.15, 95% CI 1.03, 1.28) and treatment dose (AOR 0.96, 95 CI 0.92, 0.99) were significantly associated with poor glycemic control. Prevalence of poor glycemic control was high. Patient’s age, history of treatment discontinuation and dose of treatment were the significant contributing factors to poor glycemic control. These need to be addressed to attain the objective of adequate glycemic control.

## Introduction

Diabetes Mellitus (DM) is a metabolic condition marked by high level of blood glucose^1,2^. Diabetes is one of the common non communicable diseases and the prevalence is high. DM is the potential for lifelong chronic complications^3^. DM is a global public health problem, that causes around five million deaths per year^4^. In 2013, there were 382 million individuals living with diabetes worldwide, with a prevalence rate of 8.3%. Type one diabetes mellitus (T1DM) is the most common endocrine-metabolic disorder in children and adolescents worldwide, with a prevalence of 190 per 100,000 among school aged children in USA and an annual incidence ranging from 1.7 per 100,000 in China to 40 per 100,000 in Finland^5^. Similarly diabetes affects an estimated 1.1 million children under the age of 20 worldwide, with 132 600 new cases diagnosed each year, representing a 3% annual increase^6^. The global prevalence of DM have increased considerably, specifically in Sub- Saharan Africa^7^. About 80% of diabetes deaths occur in low as well as middle income countries^8,9^.

Diabetes results from elevated levels of glycaemia such as increased glucose and glycated hemoglobin (A1C), and controlling glycaemia is an integral component of the management of diabetes^10^. Glycated hemoglobin (A1C) is one of the most widely used test to diagnose DM and to monitor glycemic control^10^. Glycemic control is the primary therapeutic objective for the prevention of target organ damage and other complications^11^. Poor glycemic control constitutes a major public health issue and a key risk factor for the development of diabetes-related complications, diseases related healthcare expenses, lower life expectancy and quality of life^4^. Long-term uncontrolled hyperglycemia results several macro-vascular and micro-vascular complications such as neuropathy, nephropathy, retinopathy, cardiovascular diseases, amputations, and even premature death^6,12,13^. Several studies indicated that more than half of diabetic patients have poor glycemic control^12,14–16^.

According to a recent study by the Ministry of Health (MoH), patients with diabetes have suboptimal glycemic control due to insufficient training of primary health care workers, a lack of access to essential diabetes medications, technology, awareness, and a failure to properly document diabetes data^13^. According to the evidence, the main therapeutic goal for all diabetic patients is to maintain good glycemic control so as to prevent organ damage such that micro-vascular and macro-vascular complications^17^. Good glycemic control reduces the risk of diabetic related complications and death^7^. However, the majority of patients fail to achieve good glycemic control. The reasons for this are complex and multi-factorial^8^.

Patients need to adhere to medications, undertake lifestyle modifications, and monitor their blood glucose levels frequently for achieving proper glucose control^18^. As demonstrated by the Diabetes Control and Complications Trial and the Epidemiology of Diabetes Interventions and Complications study, the improvement of glycemic control in both children and adolescents with T1DM leads to a decreased risk of diabetic complications^19^. However, considering the physiological and behavioral challenges that children with T1DM, optimizing glycemic control in this age group is particularly difficult^20^. Identifying the determinants of poor glycemic control is important for early modification of diabetic related end organ damage. This also allows patients to maintain good glycemic control. Therefore, this study aimed to assess the level of glycemic control among pediatric DM patients in northwest Ethiopia.

## Methodology

### Study design, period, and area

A facility-based cross sectional retrospective cohort study design was used and this study was conducted from September 2015 to February 2018. The study was carried out at the University of Gondar Comprehensive and Specialized Referral Hospital (UoGCSRH) and Felege Hiwot Comprehensive and Specialized Referral Hospital (FHCSRH) in the Amhara Regional State, in northwest Ethiopia. The catchment population in both hospitals is expected to be five million individuals. Both hospitals provide pediatric emergency, inpatient wards, outpatient follow-up, and chronic illness Out Patient Department (OPD) services. Between 2015 and 2018, 322 and 301 pediatric DM patients were followed at UoGCSRH and FHCSRH, respectively.

### Study subjects and sampling procedures

All pediatric DM patients at UoGCSRH and FHCSRH were the source population, and all pediatric DM patients who started treatment at the both hospitals from 2015 to 2018 were the study populations. The study included pediatric DM patients’ under the age of 18 who were followed from 2015 to 2018. Pediatric DM patients, whose diagnosis and follow up dates unknown were excluded from this study.

The normalized Poisson suggested method of Signori (1991) was used to determine sample size. Based on the approach, using a 95% confidence level and 90% power, the final sample size used for this study was 389. Patients were selected using simple random sampling method. Based on the study period, a patient record numbers (MRN) were selected from the logbooks. The estimated samples were allocated proportionally to the two hospitals, 201 samples from UoGCSRH and 188 from FHCSRH and simple random sampling by table of random numbers was used to select the samples.

### Operational definition

*Glycemic control* Is a medical term referring to levels of blood glucose in a person with diabetes mellitus which were categorized by American Diabetic Association (ADA) recommendation based on the value of glycosylated hemoglobin^21,22^.

*Good glycemic control* fasting blood glucose of 70 ± 130 mg/dl, HbA1c was below 7%^11,22^.

*Poor glycemic control* fasting blood glucose of < 70 mg/dl and > 130 mg/dl, HbA1c was found to be more or equal to 7%^11,22^.

*Fasting blood sugar* Blood glucose measured from venous blood after 8 h of overnight fasting or longer^21,23^.

### Severity of DKA

*Mild* Plasma glucose > 250 mg/dl, urine ketone positive, Arterial pH of 7.25–7.30 and Alteration in sensorium become alert^24^.

*Moderate* Plasma glucose > 250 mg/dl, urine ketone positive, Arterial pH of 7.00–< 7.24 and Alteration in sensorium become alert or drowsy^24^.

*Sever* Plasma glucose > 250 mg/dl, urine ketone positive, Arterial pH of < 7.00 and Alteration in sensorium become stuporous or comatose^24^.

### Nutritional status

*Wasting/thinness* is defined as children with weight—for—height Z-score (WAZ) < − 2 SD^25,26^.

*Stunting* is defined children with height—for-age Z-score (HAZ) <  − 2 SD^25,26^.

*Underweight* defined as the children with weight-for-age Z-score (WAZ) <  − 2 SD^25,26^.

*Anemic status* Anemic (hemoglobin < 11.0 g/dl), not anemic (hemoglobin ≥ 11.0 g/dl)^25,26^.

### Potassium level

*Hyperkalaemia* serum K +  > 5.5 mEq/l^27,28^.

*Normal* serum K + 3.5–5.0 mEq/l^27,28^.

*Hypokalaemia* is defined as a plasma potassium level less than 3.5 mmol/l^27,28^.

### Sodium level

*Hyponatraemia* is defined as serum sodium concentration < 135 mmol/l^29^.

*Normal* (135–145 mEq/l)^29^.

*Hypernatremia* serum sodium concentration > 145 mEq/l^29^.

*Pediatric age group* age less than 19 years old.

*Treatment discontinue* skipping treatments a minimum of one day including skipping of even single dose of treatment.

### Data collection procedures and quality assurance

The study was based on secondary data gathered using a check-list from patients’ charts March 4 and May 11, 2020. The data extraction check-list was developed after reviewing the patient charts and different literature. The extraction check-list included socio-demographic and clinical variables. The outcome variable was glycemic control, and the independent variables were socio-demographic characteristics (age, gender, residence), medical, nutritional, and measurement variables (type of DM, family history, severity of DKA at diagnosis, blurring of vision, foot ulcer, sodium level, potassium level, infection, wasting/thinness, stunting, underweight, RBS, and FBS).

The data were collected by six BSc nurses. Two supervisors were selected to supervise every activity of the data collection process. A 5% preliminary assessment of charts was performed at the two hospitals to verify the data quality. Before the actual data collection, data collectors and supervisors received one-day training on the content of the data extraction form and how to collect the data. Completeness of the data was checked on each day of data collection by supervisors, and the investigators had a daily meeting was held to review challenges and strengths that occurred during data collection.

### Data processing and analysis

For data entry, Epi-data version 4.6 was used. The data was cleaned, coded and checked using SPSS version 25. For nutritional measurement, the WHO Anthro version 3.2.2 for under-five children and Anthro plus version 1.0.4 for adolescents were used to categorize patients based on their nutritional status. Missing data management was done using R-studio software. The method of missing data management was the imputation method, which is flexible and applied for missing completely at random (MCAR) and missing at random (MAR). Descriptive statistics were carried out by texts, tables, and graphs. Final statistical analysis was done by Stata version 16. Binary logistic regression was used to assess the association between dependent and independent variables. In bi-variable analysis, variables with p-value of < 0.25 were entered into multivariable binary logistic regression. In multivariable analysis, p-value < 0.05 was used to determine statistical significance and interpretation was made by adjusted odds ratio (AOR) and with their respective 95% confidence intervals.

### Ethical approval and consent to participate

Ethical approval was obtained from Institutional Review Board (IRB) of institution of public health at University of Gondar. All methods were performed in accordance with the relevant guidelines and regulations. Informed consent was waived by the Institutional Review Board (IRB) of institution of public health at University of Gondar due to the retrospective nature of the study.

## Result

### Socio demographic characteristics of patients

A total of 389 patients were included in the study. More than half 215 (55.27%) of patients were female, and a nearly equal proportion, 198 (50.90%) and 191 (49.10%) of patients were from rural and urban areas respectively. The mean age of patients was 11 years (± 4 SD).

### Clinical/medical characteristics of patients

Most 316 (81.23%) of the patients were newly diagnosed and the rest were known DM. regarding family history, below one third (27.25%) of patients had family history of DM. Around 246 (63.24%) of patients had comorbidity with infection (Table1). The mean duration of follow up was 3.5 (± 0.06 SD) years. The average FBS was 272.6 (± 80.8 SD) and the average RBS was 500.9 (± 100.9 SD). Regarding the anemia status of patients, 18 (4.63%) of patients were anemic. About 26 (6.7%) of patients had foot ulcers and 41 (10.5%) of them had blurred of vision. Almost all, 368 (94.6%) of pediatric patients had DKA at the diagnosis of DM. During the follow-up, 79 (20.3%) of patients had history of treatment discontinuation.

**Table 1:** Clinical/medical characteristics of pediatric DM patients at University of Gondar and Felege Hiwot Specialized and Referral Hospitals, 2020.

Variables	Category	Frequency	%
Type of DM	Newly diagnosed	316	81.23
	Known	73	18.77
Family history	No	283	72.75
	Yes	106	27.25
Severity of DKA at diagnosis	Mild	157	42.66
	Moderate	101	27.45
	Sever	110	29.89
Blurred vision	No	348	89.46
	Yes	41	10.54
Foot ulcer	No	363	93.32
	Yes	26	6.68
Sodium level	Normal	83	21.34
	Hyponatremia	272	69.92
	Hypernatremia	34	8.74
Potassium level	Normal	238	61.18
	Hypokalemia	115	29.56
	Hyperkalemia	36	9.25
Infection	No	143	36.76
	Yes	246	63.24
Wasting/thinness	Normal	145	37.28
	Wasted/thin	244	62.72
Stunting	Normal	191	49.10
	Stunted	198	50.90
Underweight	No	255	65.55
	Yes	134	34.45
Anemia	No	371	95.37
	Yes	18	4.63

### Magnitude of poor glycemic control and its determinants

The prevalence of poor glycemic control among pediatric patients was 39.3% (95% CI 34.6, 44.3).

Variables that passed in bi-variable analysis were entered into multivariable binary logistic regression analysis. In multivariable analysis, history of treatment discontinuation, age of patients, and dose of treatment that patients took were significantly associated with poor glycemic control. Patients who discontinued their treatment during follow up were 2.42 times (AOR 2.42, 95% CI 1.25, 4.69) more likely to have a poorly controlled blood glucose level compared to those who took their treatment properly, keeping other variables constant. Regarding the age of patient, a one year increasing in the age of the patient increases the risk of poor glycemic control by 1.15 times (AOR 1.15, 95% CI 1.03,1.28) the effect of other variables kept constant. The other significant predictor of poor glycemic control was dose of treatment; therefore those patients who increased the dose of treatment by a unit were 0.96 less- likely to have poor glycemic control (decreased by 4%) (AOR 0.96, 95 CI 0.92, 0.99) (Table 2).

**Table 2:** Multivariable logistic regressions of factors associated with poor glycemic control among pediatric DM patients at University of Gondar and Felege Hiwot Specialized and Referral Hospitals, 2020.

Variables	Category	Level of glycemic control		COR	AOR (95% CI)
		Poor	Good		
Infection	No	46	97	1	1
	Yes	107	139	1.62 (1.05, 2.50)*	1.88 (0.93, 3.80)
Wasting/thinness	No	62	83	1	1
	Yes	91	153	0.79 (0.52, 1.21)	0.57 (0.29, 1.14)
Severity of DKA	Mild	33	61	1	1
	Moderate	22	18	2.26 (1.06, 4.79)*	2.08 (0.91, 4.77)
	Sever	23	28	1.52 (0.75, 3.04)	0.97 (0.45, 2.09)
History of treatment discontinuation	No	110	200	1	1
	Yes	43	36	2.17 (1.32, 3.58)*	2.42 (1.25, 4.69)*
Duration of DM	< 5 years	110	167	1	1
	≥ 5 years	43	69	0.95 (0.60, 1.48)	1.16 (0.59, 2.29)
RBS		–	–	1.001 (0.99, 1.003)	1.003 (0.99, 1.01)
Age		–	–	1.02 (0.98, 1.07)	1.15 (1.03, 1.28)*
Dose of treatment		–	–	0.99 (0.97, 1.02)	0.96 (0.92, 0.99)*

NB: *Indicates statistical significant variables at P-value of < 0.05.

## Discussion

Maintaining adequate level of blood glucose is the goal for all methods of DM therapy^30^. This study was aimed to assess determinants of poor glycemic control among pediatric DM patients in northwest Ethiopia.

The finding of this study showed that the magnitude of poor glycemic control among pediatric DM patient was 39.3% (95% CI 34.6, 44.3). This figure is lower than studies conducted in Egypt (45.8%), Niger (55%), Amhara region (55.32%), Zambia (61.3%), Gondar (61.4%), western Ethiopia (64.1%), Tikur Anbessa Specialized Hospital (80%) ,Sudan Khartoum (76%)^2,3,15,18,30–33^. The discrepancy in proportion of people with poor glycemic control may be due to the difference in sample size, scio-demographic characteristics and availability of diabetic treatment services. The other possible reason could be that different countries have different nutritional habits, living standards, and knowledge of preventive and treatment measures^31^. Poor glycemic control can be attributed to lack of well-structured diabetes education programs for children and families^19^.

Concerning determinants of poor glycemic control, medication adherence is important for patient glycemic control. In this study those patients who discontinued their treatment were more likely to have poor glycemic control. This finding is supported by studies conducted in Japan, Singapore, and Kenyatta National Hospital^34–36^. This might be because patients in our setting didn’t have access to health insurance and were unable to afford the treatment’s cost. Treatment discontinuation may be followed by increased glucose levels and other complications, including acute and chronic complications, which finally lead to poor control of DM. Therefore, medication adherence is critical for better control of the patients’ glycemic level.

In this study age of the patient was significantly associated with poor glycemic control. As a result, if increasing the age of children by one year, the patient is more likely to develop poor glycemic control. This finding was supported by studies conducted in Amhara region, Egypt, and Bulgaria, which showed that patients with poor control had a significantly higher mean age^2,17,37^. The possible reason is due to the fact that this study includes pediatric patients which also include adolescents and they are more prone to poor diabetic control because of pubertal stress or may not be safely follow their treatment. The physiological and hormonal changes that occur during puberty like increase in adiposity and insulin resistance can be considered as another factor^19^. The other reason is that adhere to a diabetes regimen is difficult particularly for young children. This leads to frequent child hospitalizations, further medical complications and poor glycemic control^17,33^.

Even though there is no significance association between poor glycemic control and having infection as comorbidity in this study, comorbidity with infection is risky for poor glycemic control. The study conducted in Kenya and Jimma have found that patients with comorbidity were significantly associated and patients with infection were more likely to have poor glycemic control^13,23^. This study indicated that duration of DM was not significantly associated with poor glycemic control. This is similar with studies conducted in Tanzania, and India, which found that the duration of DM was not a significant predictor of poor glycemic control^6,11^. But, studies conducted in Amhara region, Shanan Gibe, Egypt, Jimma, and Nekemte showed that duration of DM was significantly associated with poor glycemic control^2,8,17,23,31^. This might be due to increased duration of the disease process, decreased insulin production, incidence of diabetes complications and progressive impairment of insulin secretion^8,38,39^. The other reason is that as duration of DM increases, patients usually become negligent of their medication adherence and glycemic control^2^.

In this study, treatment dose was significantly and negatively associated with poor glycemic control. The risk of poor glycemic control was reduced by increasing treatment dose by one IU until it reached recommended dose. However, the study conducted in Tanzania indicated that dose of treatment is not a significant predictor of poor glycemic control^6^. The discrepancy might be due to the difference in study participant, sample size and setting.

### Strength and limitation of study

Though this study did its best to identify determinants of poor glycemic control among pediatric DM patients in the study setting, it was not free from some limitations. This study has missed variables important to explain poor glycemic control and limitation by the method itself.

## Conclusion

The magnitude of poor glycemic control among pediatric DM patients was high. Patients’ age, history of treatment discontinuation and dose of treatment were determinants for poor glycemic control. This finding gives insight on important predictors that need to be addressed and intervention required for better management of pediatric DM and finally to reach the diabetic management goal which is achieving good glycemic control.

## Data Availability

Data will be available upon request from the corresponding author.

## Abbreviations

AOR: Adjusted odds ratio
COR: Crude odds ratio
CI: Confidence interval
DKA: Diabetic ketoacidosis
DM: Diabetes mellitus
FHCSRH: Felege Hiwot comprehensive and specialized referral hospital
IDF: International diabetes federation
IRB: Institutional review board
MAR: Missing at random
MCAR: Missing completely at random
MRN: Medical record number
OPD: Out patient department
RBS: Random blood sugar
SD: Standard deviation
T1DM: Type 1 diabetes mellitus
UoGSRH: University of Gondar comprehensive and specialized referral hospital

## References

[1] Yosef T, Nureye D, Tekalign E (2021). Poor glycemic control and its contributing factors among type 2 diabetes patients at Adama hospital medical college in East Ethiopia. Diabet. Metab. Syndr. Obes. Targets Ther..

[2] Feleke BE, Feleke TE, Kassahun MB, Adane WG, Fentahun N, Girma A (2021). Glycemic control of diabetes mellitus patients in referral hospitals of Amhara Region, Ethiopia: A Cross-Sectional Study. BioMed Res. Int..

[3] Tekalegn Y, Addissie A, Kebede T, Ayele W (2018). Magnitude of glycemic control and its associated factors among patients with type 2 diabetes at Tikur Anbessa Specialized Hospital, Addis Ababa, Ethiopia. PLoS ONE.

[4] Fiseha T, Alemayehu E, Kassahun W, Adamu A, Gebreweld A (2018). Factors associated with glycemic control among diabetic adult out-patients in Northeast Ethiopia. BMC. Res. Notes.

[5] Ngwiri T, Were F, Predieri B, Ngugi P, Iughetti L (2015). Glycemic control in Kenyan children and adolescents with type 1 diabetes mellitus. J. Endocrinol..

[6] McLarty RP, Alloyce JP, Chitema GG, Msuya LJ (2021). Glycemic control, associated factors, acute complications of Type 1 Diabetes Mellitus in children, adolescents and young adults in Tanzania. Endocrinol. Diabet. Metab..

[7] Gebermariam AD, Tiruneh SA, Ayele AA, Tegegn HG, Ayele BA, Engidaw M (2020). Level of glycemic control and its associated factors among type II diabetic patients in debre tabor general hospital, northwest Ethiopia. Metab. Open..

[8] Yigazu DM, Desse TA (2017). Glycemic control and associated factors among type 2 diabetic patients at Shanan Gibe Hospital, Southwest Ethiopia. BMC Res. Notes..

[9] Djonou C, Tankeu AT, Dehayem MY, Tcheutchoua DN, Mbanya JC, Sobngwi E (2019). Glycemic control and correlates in a group of sub Saharan type 1 diabetes adolescents. BMC. Res. Notes.

[10] Elizabeth Selvin P (2021). Measurements of Glycemic Control in Diabetes Mellitus.

[11] Khattab M, Khader YS, Al-Khawaldeh A, Ajlouni K (2010). Factors associated with poor glycemic control among patients with type 2 diabetes. J. Diabetes Compl..

[12] Gebreyohannes EA, Netere AK, Belachew SA (2019). Glycemic control among diabetic patients in Ethiopia: A systematic review and meta-analysis. PLoS ONE.

[13] Nduati NJ, Simon K, Eva N, Lawrence M (2016). Factors associated with glycemic control among type 2 diabetes patients attending Mathari National Teaching Hospital, Nairobi Kenya. J. Endocrinol. Diabetes..

[14] Kibirige D, Akabwai GP, Kampiire L, Kiggundu DS, Lumu W (2017). Frequency and predictors of suboptimal glycemic control in an African diabetic population. Int. J. Gen. Med..

[15] Musenge EM, Manankov A, Mudenda B, Michelo C (2014). Glycaemic control in diabetic patients in Zambia. Pan Afr. Med. J..

[16] Suyagh M, Farah D, Farha RA (2015). Pharmacist’s knowledge, practice and attitudes toward pharmacovigilance and adverse drug reactions reporting process. Saudi Pharm. J..

[17] Mohammad HA, Farghaly HS, Metwalley KA, Monazea EM, Abd El-Hafeez HA (2012). Predictors of glycemic control in children with Type 1 diabetes mellitus in Assiut-Egypt. Indian J. Endocrinol. Metab..

[18] Ufuoma C, Godwin YD, Kester AD, Ngozi JC (2016). Determinants of glycemic control among persons with type 2 diabetes mellitus in Niger Delta. Sahel Med. J..

[19] Al Zahrani AM, Al SA (2019). Glycemic control in children and youth with type 1 diabetes mellitus in Saudi Arabia. Clin. Med. Insights Endocrinol. Diabetes.

[20] Dumrisilp T, Supornsilchai V, Wacharasindhu S, Aroonparkmongkol S, Sahakitrungruang T (2017). Factors associated with glycemic control in children and adolescents with type 1 diabetes mellitus at a tertiary-care center in Thailand: A retrospective observational study. Asian Biomed..

[21] Association A (2020). American diabetes association standards of medical care in diabetes-2020. Diabetes Care.

[22] Assessment G (2021). Glycemic targets: Standards of medical care in diabetes 2021. Diabetes Care.

[23] Mamo Y, Bekele F, Nigussie T, Zewudie A (2019). Determinants of poor glycemic control among adult patients with type 2 diabetes mellitus in Jimma University Medical Center, Jimma zone, south west Ethiopia: A case control study. BMC Endocr. Disord..

[24] Umpierrez GE, Murphy MB, Kitabchi AE (2002). Diabetic ketoacidosis and hyperglycemic hyperosmolar syndrome. Diabetes Spectr..

[25] Tesema GA, Worku MG, Tessema ZT, Teshale AB, Alem AZ, Yeshaw Y, Liyew AM (2021). Prevalence and determinants of severity levels of anemia among children aged 6–59 months in sub-Saharan Africa: A multilevel ordinal logistic regression analysis. PLoS ONE.

[26] Shrestha A, Bhusal CK, Shrestha B, Bhattarai KD (2020). Nutritional Status of Children and Its Associated Factors in Selected Earthquake-Affected VDCs of Gorkha District, Nepal. Int. J. Pediatr..

[27] The Royal Children's Hospital Melbourne CPG.

[28] Raza M, Kumar S, Ejaz M, Azim D, Azizullah S, Hussain A (2020). Electrolyte imbalance in children with severe acute malnutrition at a tertiary care hospital in Pakistan: a cross-sectional study. Cureus.

[29] Robba C, Rosenstein I, Bilotta F (2021). Fluid and Electrolyte Therapy A Chapter in Core Concepts of Pediatrics.

[30] Fasil A, Biadgo B, Abebe M (2019). Glycemic control and diabetes complications among diabetes mellitus patients attending at University of Gondar Hospital, Northwest Ethiopia. Diabetes Metab. Syndr. Obes. Targets Ther..

[31] Oluma A, Abadiga M, Mosisa G, Etafa W (2021). Magnitude and predictors of poor glycemic control among patients with diabetes attending public hospitals of Western Ethiopia. PLoS ONE.

[32] Hamed ZSS, Gawaly AM, Abbas KM, El Ahwal LM (2017). Epidemiology of infection as a precipitating factor for diabetic ketoacidosis at Tanta University Hospital. Tanta Med. J..

[33] Taha Z, Eltoum Z, Washi S (2018). Predictors of glucose control in children and adolescents with type 1 diabetes: Results of a cross-sectional study in Khartoum, Sudan. Open Access Macedonian J. Med. Sci..

[34] Tominaga Y, Morisky DE, Mochizuki M (2021). A cross-sectional study clarifying profiles of patients with diabetes who discontinued pharmacotherapy: Reasons and consequences. BMC Endocr. Disord..

[35] Lin L-K, Sun Y, Heng BH, Chew DEK, Chong P-N (2017). Medication adherence and glycemic control among newly diagnosed diabetes patients. BMJ Open Diabetes Res. Care.

[36] Waari G, Mutai J, Gikunju J (2018). Medication adherence and factors associated with poor adherence among type 2 diabetes mellitus patients on follow-up at Kenyatta National Hospital, Kenya. Pan Afr. Med. J..

[37] Archinkova M, Konstantinova M, Savova R, Iotova V, Petrova C, Kaleva N (2017). Glycemic control in type 1 diabetes mellitus among Bulgarian children and adolescents: The results from the first and the second national examination of HbA1c. Biotechnol. Biotechnol. Equip..

[38] Li J, Chattopadhyay K, Xu M, Chen Y, Hu F, Chu J (2018). Glycaemic control in type 2 diabetes patients and its predictors: a retrospective database study at a tertiary care diabetes centre in Ningbo, China. BMJ Open.

[39] Pamungkas RA, Mayasari A, Nusdin N (2017). Factors associated with poor glycemic control among type 2 diabetes mellitus in Indonesia. Belitung Nurs. J..

